# Manufacturing a First Upper Molar Dental Forceps Using Continuous Fiber Reinforcement (CFR) Additive Manufacturing Technology with Carbon-Reinforced Polyamide

**DOI:** 10.3390/polym13162647

**Published:** 2021-08-09

**Authors:** Roland Told, Gyula Marada, Szilard Rendeki, Attila Pentek, Balint Nagy, Ferenc Jozsef Molnar, Peter Maroti

**Affiliations:** 1 3D Printing and Visualization Centre, University of Pecs, Boszorkány Street 2, 7624 Pécs, Hungary; told.rland@pte.hu (R.T.); pentek.attila@pte.hu (A.P.); 2 Clinical Centre, Department of Dentistry, Oral and Maxillofacial Surgery, University of Pecs, Dischka Győző Street 5, 7621 Pécs, Hungary; marada.gyula@pte.hu; 3 Medical Simulation Education Centre, Medical School, University of Pecs, Szigeti Road 12, 7624 Pécs, Hungary; rendeki.szilard@pte.hu (S.R.); fecni1990@gmail.com (F.J.M.); 4 Clinical Centre, Department of Anesthesiology and Intensive Therapy, University of Pecs, Ifjúság Roud 13, 7624 Pécs, Hungary; balintjanosnagy@gmail.com

**Keywords:** 3D printing, additive manufacturing, dental forceps, CFR (continuous fiber reinforcement), fatigue test, mechanical testing, composite, carbon, scanning electron microscopy

## Abstract

3D printing is an emerging and disruptive technology, supporting the field of medicine over the past decades. In the recent years, the use of additive manufacturing (AM) has had a strong impact on everyday dental applications. Despite remarkable previous results from interdisciplinary research teams, there is no evidence or recommendation about the proper fabrication of handheld medical devices using desktop 3D printers. The aim of this study was to critically examine and compare the mechanical behavior of materials printed with FFF (fused filament fabrication) and CFR (continuous fiber reinforcement) additive manufacturing technologies, and to create and evaluate a massive and practically usable right upper molar forceps. Flexural and torsion fatigue tests, as well as Shore D measurements, were performed. The tensile strength was also measured in the case of the composite material. The flexural tests revealed the measured force values to have a linear correlation with the bending between the 10 mm (17.06 N at 5000th cycle) and 30 mm (37.99 N at 5000th cycle) deflection range. The findings were supported by scanning electron microscopy (SEM) images. Based on the results of the mechanical and structural tests, a dental forceps was designed, 3D printed using CFR technology, and validated by five dentists using a Likert scale. In addition, the vertical force of extraction was measured using a unique molar tooth model, where the reference test was carried out using a standard metal right upper molar forceps. Surprisingly, the tests revealed there to be no significant differences between the standard (84.80 N ± 16.96 N) and 3D-printed devices (70.30 N ± 4.41 N) in terms of extraction force in the tested range. The results also highlighted that desktop CFR technology is potentially suitable for the production of handheld medical devices that have to withstand high forces and perform load-bearing functions.

## 1. Introduction

In recent years, additive manufacturing (AM) technology has become an undoubtedly decisive tool in the healthcare industry. It is widely used in model creation and visualization [1], simulator development [2], preoperative planning [3], prototyping, and the small-series production of medical devices such as implants [4], prosthetics and orthotics [5,6], and laboratory equipment, and can also support tissue engineering processes [7]. Furthermore, it can strongly enhance and support patient–doctor communication, and serve patient education purposes as well [8].

3D printers are used on a daily basis in dental care. Several dentistry-related applications are well described and deeply studied, since the technology provides a relatively cost-effective, fast, easy-to-use, and customizable solution for healthcare professionals. Metal dental implants, screws, and abutments can be produced using DMLS (direct metal laser sintering) technology, mainly from metal alloys such as Co-Cr alloy steel or titanium [9,10]. PolyJet™ and FFF (fused filament fabrication) technologies are mainly used for creating replica teeth, skulls, and mandibular or maxillary models in order to perform preoperative planning [11]. The most commonly used devices are desktop SLA (stereolithography) 3D printers, and their applications include model fabrication, surgical guide or mold printing, and complex prototyping processes [9,10]. Additive manufacturing is also essential in orthodontics, restorative dentistry, and endodontics [12]. Despite the fact that dental applications are strongly rely on handheld devices, only a few previous studies are available concerning the use of 3D printing in instrument development and production. Recently, surgical devices printed for long-duration space missions have been reported using FFF technology and ABS (acrylonitrile butadiene styrene) material [13,14,15], but dentistry-related applications have not yet been reported. This lack of studies potentially indicates that desktop FFF 3D printing technology has serious disadvantages in terms of mechanical and structural stability, due to the anisotropic material characteristics of the end-products, which mainly occur through the use of PLA (polylactic acid) and ABS.

CFR (continuous fiber reinforcement) additive manufacturing has outstanding mechanical properties compared to FFF. The addition of carbon fibers can increase both tensile strength and the modulus of elasticity [16]. It has been observed that parts and models created using carbon-reinforced polyamide can be created with high precision, and are comparable with molded or even metal parts; however, further investigations are essential in order to better understand their structural characteristics, and to avoid weak interlayer connections [17,18].

The aim of this study was to explore the possibilities CFR AM technology can provide in the field of handheld medical device development and production. The mechanical characterization focused on fatigue testing, in order to determine the usability in load-bearing applications. Structural analysis was performed to explore the mechanisms behind the observed results. The results were compared with neat PLA models printed with an FFF 3D printer. Moreover, the goal was to design, fabricate, and test a functional handheld device—in this case, a first upper molar dental extractor, since this medical instrument must be characterized with excellent mechanical properties and the proper level of durability, in order to withstand outstandingly high forces.

## 2. Materials and Methods

### 2.1. 3D Printing Parameters and Fabrication of Test Specimens

In order to fabricate the PLA control test specimens, a Craftbot Plus 3 (Craftbot Ltd., Salgótarjáni rd. 12–14, Budapest, Hungary) 3D printer was used, with a 0.4-mm nozzle, 0.2-mm layer height, and 100% infill density. A 245 °C primary extruder temperature and 110 °C heated-bed temperature provided the necessary amount of heat for fabrication. Neat PLA was utilized, (Herz Hungária Kft., Pesti rd. 284, Üllő, Hungary), with a filament diameter of 1.75 mm and a white color. The specimens were sliced using CraftWare™ software (Craftbot Ltd., Salgótarjáni rd. 12–14, Budapest, Hungary).

To produce the composite-based test specimens and the dental extractor, a Markforged X7 continuous fiber reinforcement (CFR)) 3D printer (Markforged, 480 Pleasant St, Watertown, MA, USA) was applied using Onyx carbon fiber composite (Markforged Co.). The specimens were sliced on the www.eiger.io, accessed on 30 July 2021 (Markforged 480 Pleasant, St Watertown, MA, USA) webpage. Onyx—which is polyamide filled with micro carbon fiber with isotropic direction properties—served as a base material. The printing was conducted in accordance with the manufacturing settings with maximum carbon fiber filling. The layer height was 0.125 mm and had a triangular fill pattern. The infill density of the fibers was 37%, and the number of wall layers was set to 2. The sample specimen printing orientation was “X” in case of both FFF and CFR printing.

To create the custom tooth model for testing the forceps, a Prusa I3 MK3 (Prusa Research a.s, Partyzánská 188/7a, Praha 7, Czech Republic) desktop FFF 3D printer was used with polyethylene terephthalate glycol (PETG) (Devil Design Sp. J. Zwirki I Wigury, Poland). The diameter of the filament was 1.75 mm. The layer height was 0.1 mm with 50% infill density, and in accordance with the melting temperature of PETG, the extruder was set to 230 °C and the bed heating was 80 °C. The slicer program was the default PrusaSlicer 2.3.

### 2.2. Material Testing

#### 2.2.1. Flexural Fatigue Test

**Figure 1:**
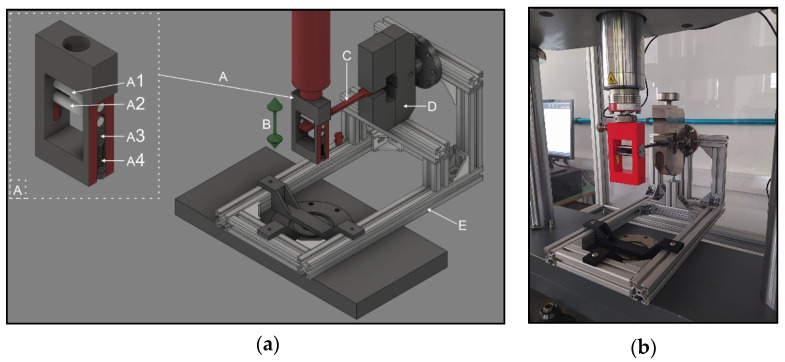
(**a**) The models used in the setup of the flexural fatigue test measurement. (**a**) Special gripper; (**a1**) fixed roller; (**a2**) moving roller; (**a3**) spring; (**a4**) adjusting screw; (**b**) moving direction; (**c**) specimen; (**d**) screw grip; (**e**) support frame. (**b**) The assembled device for the flexural fatigue test measurement.

In case of the PLA and the carbon composite test specimens, firstly, flexural fatigue tests were carried out using a ZwickRoell e/m actuator material tester (ZwickRoell, 89079, August-Nagel-Straße 11, Ulm, Germany) with a 5-kN load cell. The type of specimen was A1 from the ISO 527–2:2012 standard. To perform the tests, a special gripper was designed that aimed to correct the increment in the cross-section due to deflection (Figure 1, Supplementary Materials). For the other end of the gripper of the test bar, a holder was fabricated and secured in horizontal position throughout the entire test procedure. Using the test specimens, a pulsating pressure test was performed, where the starting force was 0 N. The deflection was 10 mm, 15 mm, 20 mm, 25 mm, 30 mm, 35 mm, 40 mm, 45 mm, and 50 mm, respectively, and it was performed until fracture, with all specimens being measured once. The testing frequency was 2 Hz, and the move was sinusoidal. In the case of the Onyx–carbon composite the tests were carried out until 20,000 cycles, and the PLA was tested until fracture in all cases. The permanent deformation was also measured. Deflection was determined with a Mitutoyo CD-15APX digimatic caliper (Mitutoyo, Sakado, Takatsu-ku, Kawasaki, Kanagawa, Japan), and the cross-section change was measured with a Mitutoyo 102–707 micrometer. The measurement was carried out on all fatigue specimens.

#### 2.2.2. Torsion Fatigue Test

**Figure 2:**
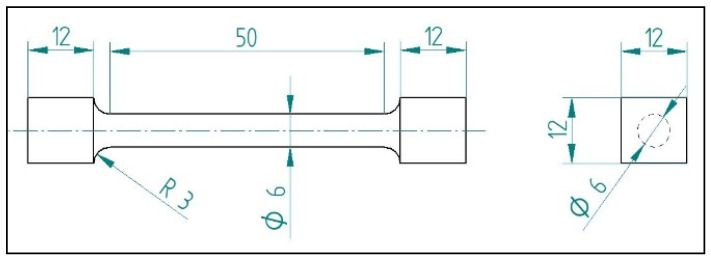
Schematic of torsion fatigue test specimens.

The torsion fatigue tests were performed with a ZwickRoell Z5.0 biaxial material tester (ZwickRoell, 89079, August-Nagel-Straße 11, Ulm, Germany). The diameter of the probe was 6 mm, the parallel length was 50 mm, and the ends of the specimen were modified in order to fit to the biaxial clamping [19] (Figure 2). The axial preload was set to 0.1 N at the start of the test, and then the axial position was fixed. Torsion preload was not applied; the testing speed was 720°/min, and the testing frequency was 2 Hz. The specimens went under sinusoidal rotation, and were tested every 10°, between 20° and 90°, for up to 20,000 cycles in the case of the composite, or until the specimens broke in the case of PLA specimens. All specimens were measured once. During the tests, the specimens’ temperatures were monitored with a Guide B160V infrared camera (Wuhan Guide Infrared Co, Ltd, No. 6, Huanglongshan South Road, East Lake Development Zone, Wuhan, China) to avoid overheating.

#### 2.2.3. Shore D Measurements

Shore D measurements were performed on both materials, using a ZwickRoell 3131 Shore D testing device (ZwickRoell, 89079, August-Nagel-Straße 11, Ulm, Germany) with the MSZ EN ISO 868:2003 (A, D) standard. The thickness of the test specimens was 4 mm, the room temperature was 25.0 °C, and the relative humidity was 47%. Both the PLA and composite test specimens were measured 5 times.

#### 2.2.4. Tensile Test

In order to investigate the effect of fatigue tests on the flexural and torsional carbon composite test specimens, tensile tests were carried out using a ZwickRoell Z100THW universal material tester (ZwickRoell, 89079, August-Nagel-Straße 11, Ulm, Germany) with a 10-kN load cell. The preload was 0.1 MPa and the test speed was 50 mm/min, according to the ISO 527 standard.

#### 2.2.5. Scanning Electron Microscopy (SEM)

To better understand the mechanical behavior of the composite material, SEM imaging was carried out with 30× and 350× magnification using a JEOL JSM-IT500HR device (3-1-2 Musashino, Akishima, Tokyo, Japan). PLA specimens were not investigated, since previous international studies have described them in detail [20]. The first group of the SEM samples was taken from the specimens with 50-mm bending distance after 20,000 cycles of flexural fatigue testing, while the other group was produced from test specimens that broke in one testing phase. The outer layers of the objects were notched before fracture, in order to prevent further damage to their structure. Before imaging, the composite samples were coated with gold using a JEOL JFC-1300 auto fine coater.

#### 2.2.6. Upper Right First Molar Forceps Scanning, Design, and Printing

To test the CFR technology with the carbon-reinforced polyamide material, a handheld dental extractor was used as a model. The reverse engineering of an upper right first molar forceps was performed suing an Artec Space Spider industrial handheld scanner (Artec, 20 rue des Peupliers, L-2328, Luxembourg, Luxembourg). After disassembling the forceps, the object went through a surface treatment to make the glittering surfaces completely visible during the scanning. This was achieved with a Centrochem (Centrotool Kft, 1102 Budapest, Halom u. 1. Hungary) cracking examination aerosol agent. The digitization of the treated components was finished separately. From the scanning results, the point-cloud processing and mesh generation were carried out in Artec Studio 15 (Artec, 20 rue des Peupliers, L-2328, Luxembourg, Luxembourg). Repair of minor errors in the generated model and the export of the 3D-printable files were done using Blender 3D modeling software (Blender Institute B.V. Buikslotermeerplein 161, ET Amsterdam, The Netherlands).

**Figure 3:**
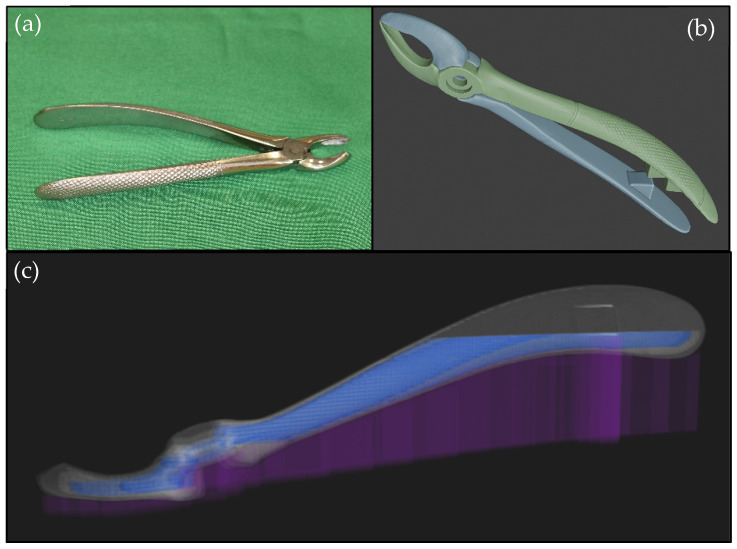
The mains steps of reverse engineering and 3D design of the forceps: (**a**) the original metal dental extractor; (**b**) the modified model in .stl format; (**c**) the sliced model ready for printing.

Due to the applied CFR printing technology, replicated objects will have some flexibility. In order to enable the forceps to exert appropriate strength and improve their usability, minor modifications were made to the geometric structure. An additional 3 mm of thickness was added to the area of the fulcrum in order to make it more resistant to load stress. The jaws are in contact at the closed state; in this way, sufficient force can be applied to carry out the extraction. Additionally, a spacer pair was placed on the inside of the forceps handles to keep the user’s finger safe when closing the instrument (Figure 3).

#### 2.2.7. Clamp Test with Biaxial Tester and Likert Scale

**Figure 4:**
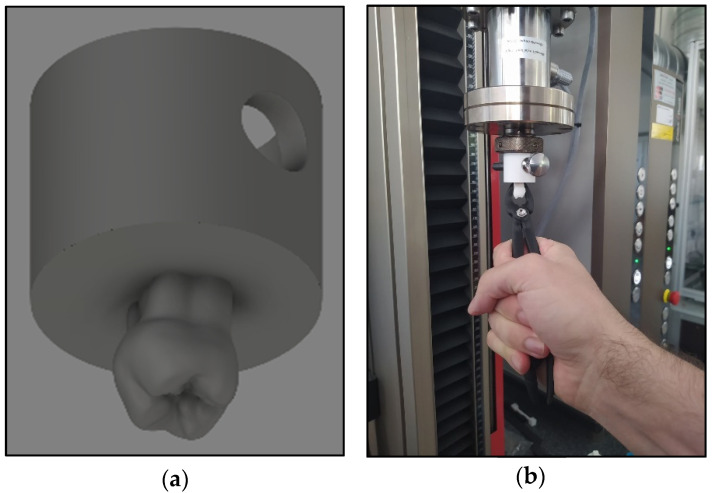
(**a**) The tooth model in .stl format; (**b**) the printed tooth model applied to the biaxial tester.

A real-size (1:1) tooth model was designed using Autodesk Fusion 360 software (111 McInnis Parkway, San Rafael, CA, USA) merged to a Zwick Z5.0 biaxial-compatible platform and printed from PETG using a Prusa I3 3D printer, (Figure 4). Five dentists with 2–20 years of experience individually tested the metal and the unique composite forceps using the 3D-printed tooth model that was applied to the testing machine (Supplementary Materials). During the test, the dentists moved their hand holding the extractor, just as they would in case of removing a real tooth, and the device measured the values of the axial force and the torque. They anonymously evaluated and compared the instruments using a Likert scale (Table A1 in Appendix A).

#### 2.2.8. Statistics and Analysis

To the measured data of the flexural and torsion tests, an upper envelope curve was fitted. The results of extraction tests were compared via two-sample *t*-test (*p* < 0.05). The statistical analysis, and curve fitting were carried out using Origin 2018 software (OriginLab Corporation, One Roundhouse Plaza, Northampton, MA, USA). The curves in case of the fatigue tests of PLA were fitted using the following function:

$$y=y_{0}+A_{1}e^{-\frac{x}{t_{1}}}+A_{2}e^{-\frac{x}{t_{2}}}$$

where *y*_0_, *A*_1_, *A*_2_, *t*_1_, and *t*_2_ are constant values, and *x* represents the number of cycles.

## 3. Results

### 3.1. Material Testing

#### 3.1.1. Flexural Fatigue Test

**Figure 5:**
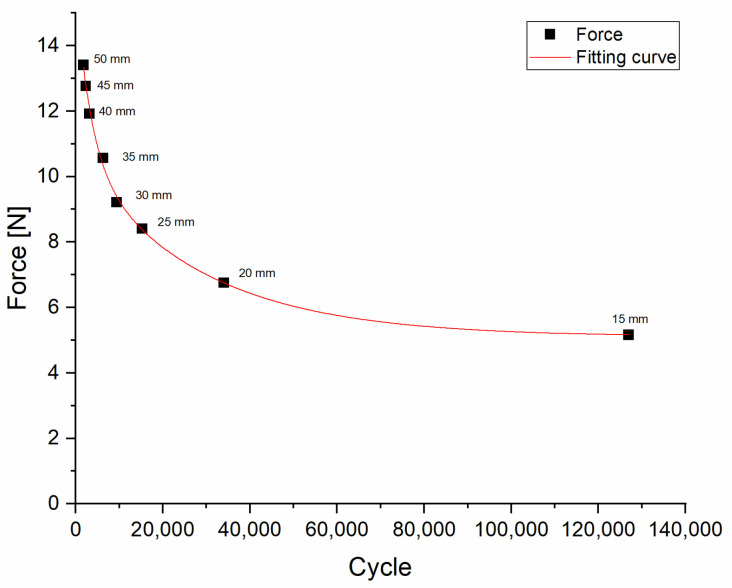
Results of the flexural fatigue test for PLA. The figure indicates the force values measured at the fracture point of each test specimen. The black squares indicate the force values (N) measured at each deflection rate (mm), with the number of cycles needed to break the test specimens.

Flexural fatigue tests were performed on both materials, in order to determine their resistance against forces that occur when the medical instrument is under tilting, bending, or flexing loads. For the PLA material, the fatigue curve corresponded to previous studies, where the characterization was carried out in detail, including the effects of different layer heights, printing speeds, and infill densities and patterns. Rotation and bending tests were also performed [20,21]. The specimen did not break during the fatigue flexural test, since it was sufficiently flexible; therefore, its flexural strength was not measured. Surprisingly, the test specimen broke only after 125,000 cycles with 15-mm deflection. (Figure 5).

**Figure 6:**
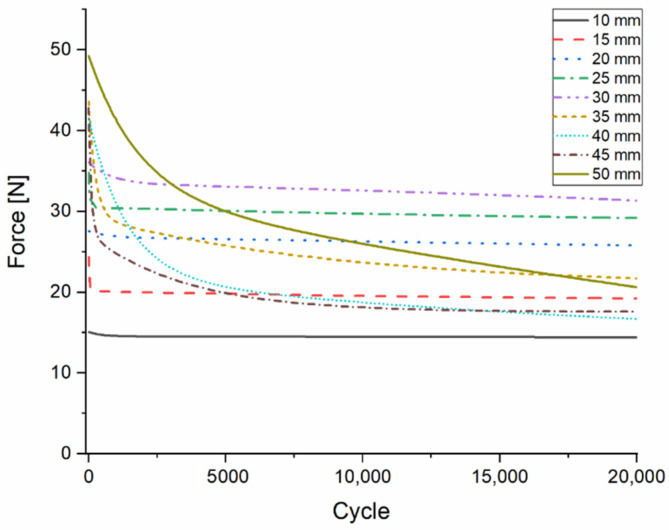
Results of the flexural fatigue test of the composite material. Force values measured at each deflection rate. The colored lines indicate the change in the force values (N), measured up to 200,000 cycles.

The Onyx composite material showed nearly constant force values between 10-mm and 30-mm deflection, with a continuous, slight decrease, measured up to 20,000 cycles. Further analysis highlights that the correlation between the bending and the measured force values was linear between 10 mm and 40 mm, but after 5000 cycles, the linearity was only observable between 10 mm and 30 mm deflection rates. At 10-mm flexion, 15.37 N was measured at the first cycle and 17.06 N at the 5000th cycle, while in the case of 30-mm deflection, 34.22 N was measured at the first cycle and 37.99 N after 5000 cycles (Figure 6). When the measurement was carried out until fracture within one test phase, the flexural strength was measured as 51.16 N and the deflection was 50.56 mm.

After performing the fatigue tests, it was observed that in the test specimens with 35-mm or higher deflection, a protrusion occurred at the point of flexion, along with a phenomenon of permanent deflection, where the highest value was 12.78 mm in the case of 45-mm deflection. The measured parameters are presented in Table 1.

#### 3.1.2. Torsion Fatigue Test

**Figure 7:**
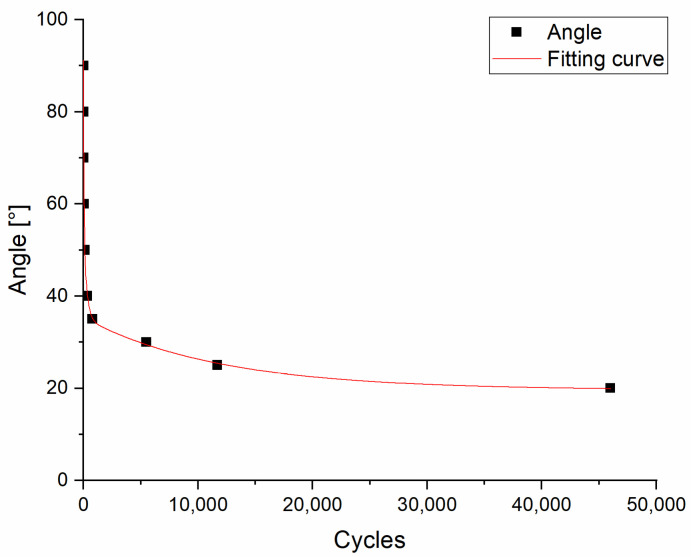
Results of torsion fatigue testing of PLA. Fracture points of test specimens with different rotation values. The black squares indicate the angle (°) and the number of cycles within which the fracture took place.

The torsion fatigue test can reveal the resistance against forces occurring when the healthcare specialists perform twisting, screwing, or scrolling movements with the handheld device. In case of the PLA test specimens, it was observed that under 35° torsion, fracture occurred within 800 cycles, but at 30° it withstood more than 5500 cycles. At 25° torsion, fracture took place at 11,700 cycles (Figure 7).

**Figure 8:**
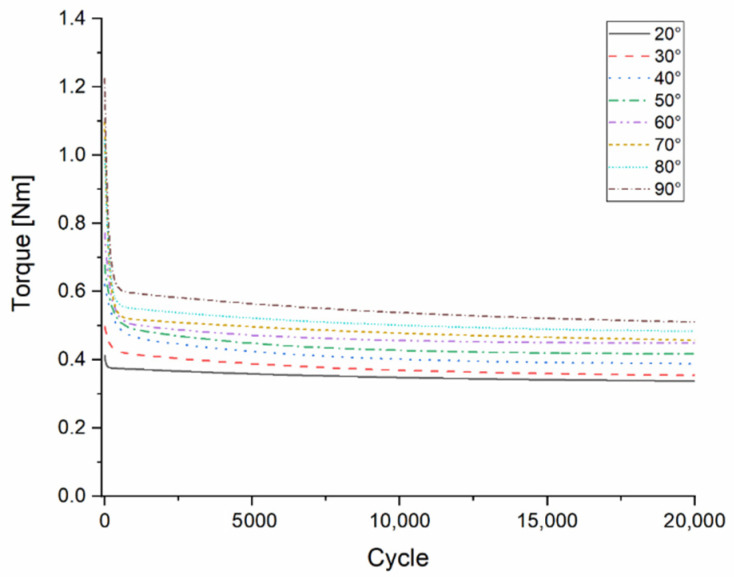
Results of torsion fatigue testing of the composite material. The colored curves show the decrease in Torque (Nm) measured at different rotation values.

The composite material at the torsion fatigue test showed similar results to bending; therefore, a classical fatigue pattern was not detected. For the test specimens after 200–300 cycles, the value of torque required to rotate the test bar was set to an approximately constant value, with a minimal decrease (Figure 8). After the test had been performed for 20,000 cycles, the test specimen’s torsional flexibility was observed, which was proportional to the maximum angle of rotation. Despite this phenomenon, the mechanical resistance of the specimens in the radial and axial directions was unchanged. In the torsion test specimens, the permanent deformations began after 40° rotation.

#### 3.1.3. Shore D Hardness Measurements

Interestingly, the Shore D measurements did not reveal significant differences (*p* < 0.05) between the PLA and the composite material. The average in the case of PLA was 79.08 ± 0.60, while it was 74.54 ± 0.59 in the case of the composite material.

#### 3.1.4. Tensile Test

**Figure 9:**
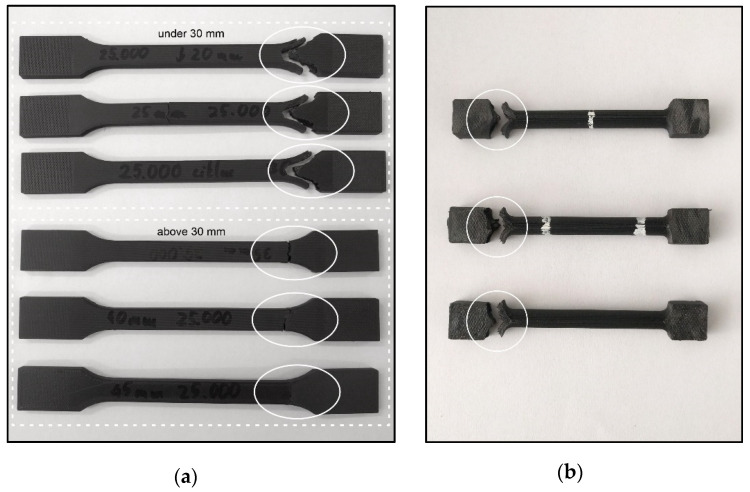
(**a**) Composite test specimens used in flexural fatigue testing, after performing the tensile strength test. The white circles indicate a “V”-like fracture pattern in the case of test specimens under 30-mm deflection; the white dotted frame represents the straight fracture line above a 30-mm deflection rate. (**b**) Composite test specimens used in torsion fatigue testing, after performing the tensile strength test. The white circles indicate the “V”-like fracture pattern.

Tensile tests were performed on the composite test specimens after flexion and torsion fatigue experiments. In accordance with the previous measurements, a significant difference was observed drawn from the results of the flexural test (two-sample *t*-test, *p* < 0.05). The average value of tensile strength was 258.30 MPa ± 10.79 MPa, and the fracture had a V shape at the rounding (Figure 9). Above 35-mm deflection, the average was 71.00 MPa ± 19.92 MPa, the fractures had a straight pattern, and the fracture locations were observed at the protrusion points (Figure 9). Moreover, the tensile test was carried out on the test specimens used in the torsion fatigue test. The results indicate that there is no major alternation between these values; the average tensile strength was 118 MPa ± 2 MPa, and the fracture had a V shape at the rounding, just like the flexural specimens (Figure 9).

### 3.2. Results of Scanning Electron Microscopy Imaging

**Figure 10:**
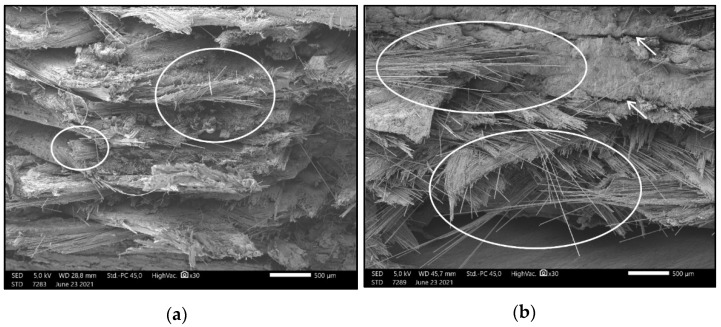
(**a**) Image demonstrating carbon fibers of nearly identical lengths (white circles); the layers are intact. (**b**) Image taken from the specimens used in the flexural fatigue test; the broken surface has cracks and hollows on it (white arrows), and the carbon fibers are elongated. The magnification was 30× in both cases.

Figure 10 was captured from a flexural test specimen’s extension zone, which refers to the horizontally upper side of the test specimen, aligned in “X” orientation. Figure 10a shows the fractured cross-section after breaking the test bar in one cycle, while Figure 10b presents the fractured cross-section after 20,000 cycles. The magnification was 30×. In the case of Figure 10a, the carbon fibers are of equal length on the fracture surface, but Figure 10b clearly demonstrates that the fibers are longer and have different lengths, varying between 500 and 2000 µm. Additionally, in this image, cracks and hollows can be observed.

**Figure 11:**
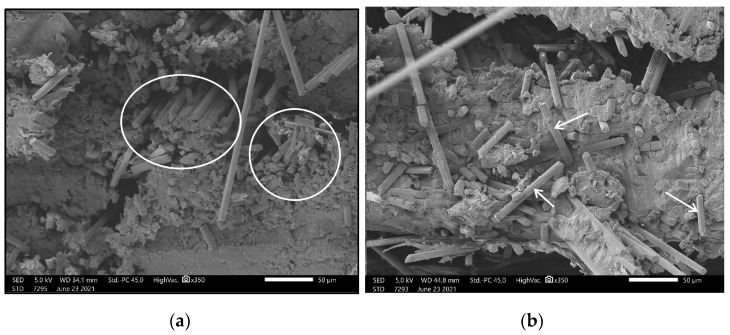
(**a**) Image showing the carbon fibers in the test specimen broken in one step; the fibers have the same length and orientation (white circle). (**b**) Image taken from the broken surface of the specimen after 20,000 flexural cycles. In this case, the carbon fibers have random orientation, and they are fragmented into small pieces (white arrows). The magnification was 350× in both cases.

Figure 11 was taken from a flexural test specimen’s compression zone. This area refers to the horizontally lower half of the test specimen, aligned in “X” orientation. Interestingly, Figure 11a shows orientated and intact carbon fibers of the equal lengths across the entire broken surface of the test specimen broken in one testing phase; meanwhile, Figure 11b presents smaller, fragmented carbon fibers after 20,000 cycles. It is notable that the carbon fiber strings do not have the same orientation—they are facing towards random directions. Both images were captured with 350× magnification.

### 3.3. Evaluation of 3D-Printed Molar Extruder

Testing was performed by professional dentists, with up to 20 years of clinical experience since their graduation. As a first step, the specialists performed the tests using a traditional metal forceps. They simulated the force of extraction on the model tooth mounted on the biaxial tester, mimicking a conventional extraction process, and after that, they also pulled the 3D-printed tooth with higher force, simulating a tougher and harder process. The measured data served as controls for the evaluation of the 3D-printed medical instrument. Following the trials with the metal extractor, the composite forceps was tested, and the dentists were asked to apply the highest possible extraction force. The results are summarized in Table 2.

Two-sample *t*-tests were performed to compare the values of the forces measured with the metal forceps and the values of forces measured with the 3D-printed composite forceps. In the case of the simulation of normal extraction, *p* = 0.15, while *p* = 0.10 when strong extraction was mimicked; therefore, there was no significant difference between the compared groups. The average force performed with the 3D-printed device was 70.30 ± 4.41 N. Surprisingly, the standard deviation was the smallest within this group. After the test, all of the dentists filled out a Likert scale. Each question was answered on a scale of 1–5, where 1 signifies “strongly disagree” or “least amount”, and 5 signifies “strongly agree” or “at the highest possible amount”. Two questions could be answered by “yes” or “no”. Furthermore, the respondents had the possibility to offer amendments and suggestions for further improvements. Overall, all specialists gave answers of 3 or 4 for the question “*How firmly could you place the forceps on the model*?”, as well as to the question “*How could you compare the similarity of the axial extraction force you used to what is necessary in a clinical environment?*”. An average of 4.4 points was given to the question that asked about the convenience of using the 3D-printed device. Only two dentists out of five would use the device in its present form for actual treatment, but with some minor modifications, all of them would use it in patient care. The detailed questions and the given answers can be found in Table A1.

## 4. Discussion

3D printing technologies are reshaping our everyday lives, including the healthcare sector. Numerous international studies have discussed in detail how additive manufacturing can support prevention, diagnosis, and medical intervention. The availability of 3D printers is continuously increasing; therefore, £d printing can be implemented in everyday clinical care. Despite the intensive research and development in the field, handheld medical devices have not been fabricated with CFR technology, nor have fatigue properties been considered previously in terms of these instruments. Similar studies have highlighted that FFF/FDM 3D printing may be suitable for the production of handheld medical devices, but detailed structural and mechanical characterization has not been carried out, and fatigue properties have not been previously investigated [13,14].

Compared to PLA, the examined carbon composite material has no classical fatigue properties; after the 100 cycles, the measured force values been remained nearly constant. Moreover, it was highlighted that CFR technology can provide sturdier and more force-resistant products compared to FFF technology. This is consistent with a previous study, where an external fixator was fabricated using this technology and the Onyx material [22]. In the case of flexural fatigue tests, 30-mm deflection can be considered to be a limit, since a significant decrease in tensile strength was measured above this value, as well as a change from “V”-shaped fracture lines to a straight pattern. This observation was also supported by the SEM images, which revealed that after a long-term fatigue test the carbon fibers broke into smaller pieces. The torsion fatigue test showed a permanent deformation beyond a 40° rotation rate, but the carbon fibers presumably remained fully intact; therefore, the axial and radial resistance did not drop during tests up to 90°—in this case, only the polyamide base material slides away. The tensile test revealed that there is no distribution between the measured values. In terms of Shore D hardness, the values correlated with previous studies [23,24], and underlined that both materials are hard enough to fabricate medical devices, but the flexibility of PLA can be a disadvantage when stability and shape retention are required.

Tooth extraction is a complex surgical process that contains gripping, twisting, and traction in order to expand the bony socket and rupture the periodontal ligament fibers. For this reason, properly geometrically shaped pliers able to reduce the applied strength needed are required. Within the limitations of this study, we measured the applied force of traction.

Based on the initial results, the first upper molar dental forceps was designed using reverse engineering methods, and printed out with a CFR desktop printer. Dental specialists critically evaluated the extractor and compared it to a traditional, metal instrument. Using a unique, real-sized tooth model, it was proven that using the forceps fabricated from the carbon composite material can provide enough force to potentially extract a real tooth in a clinical environment. Furthermore, it was highlighted that this device can be reused several times. The results were consistent with previous findings, which measured vertical tooth extraction forces in real patients, and found that the amount of force required strongly depends on root anatomy and dental status [25,26]. Based on the reports of the dentists, after some minor modifications, our device could be potentially used in real-life conditions. Their reports suggest that the elasticity should be decreased more, the jaws of the device should be more stable, and the internal profile should be more ribbed.

In accordance with previous studies, it was confirmed that additive manufacturing technologies can be useful in the manufacture of medical devices. The main target groups include facilities or missions where continuous supply is not provided—for example, medical humanitarian missions [27], remote medical sites, or even space missions [13,14]. Moreover, recently, several studies highlighted that 3D printing can be a solution in times of global crisis, such as the COVID-19 pandemic [28,29,30]. Our findings reveal that instruments printed with CFR technology can be potentially used several times, and their mechanical properties provide excellent durability. The instrument is lightweight, easy to customize, and can be fabricated without any special infrastructural needs.

Despite the mechanical characterization being carried out in detail, in the future, it will be extremely important to evaluate the potential disinfection protocols that can be used for medical instruments fabricated with 3D printers without causing major change to their material properties. This aspect is crucial in terms of reusability, and precise, critical evaluations are still needed in terms of the sterilization of these medical instruments [22,31]. Furthermore, cost-effectivity analysis should be considered; however, the availability of 3D printing technologies is continuously increasing [32]. The limitations of our study also include the relatively low number of testing volunteers. These questions will be addressed in the upcoming projects of our research team.

In the near future, after the recommended modification of the model, and after obtaining the necessary ethical approval, cadaver studies are planned in order to prepare the devices for clinical trials. We believe that the findings could be implemented in everyday patient care as well as remote medicine applications.

## 5. Conclusions

Summarizing the results of the mechanical and structural analyses and the validation process, it is highlighted that CFR technology with carbon-reinforced composite materials can be suitable for the development and production of handheld medical devices. Furthermore, these devices can be reused for several sessions and procedures. The findings of this study also aim to inspire and support further research in the field of medical device design and fabrication based on 3D printing technologies.

## Figures and Tables

**Table 1:** The table indicates the location of protrusion points compared to the middle of the test specimens (difference in mm), with the values of permanent deflection.

Bending (mm)	Thickness at Middle Point (mm)	Thickness at Flexural Point (mm)	Difference (mm)	Permanent Deflection (mm)
10	4.182	4.163	−0.019	0
15	4.174	4.18	0.006	0
20	4.154	4.093	−0.061	0
25	4.152	4.133	−0.019	0
30	4.073	4.046	−0.027	0
35	4.16	4.392	0.232	8.16
40	4.153	4.474	0.321	9.20
45	4.121	4.537	0.416	12.78
50	4.059	4.397	0.338	10.78

**Table 2:** Summary of the results of the simulation of tooth extraction.

	Metal Forceps		3D-Printed Composite Forceps
Dentist	Force of Normal Extraction of Tooth (N)	Force of Strong Extraction of Tooth (N)	Force Performed with Composite Forceps (N)
Dentist 1.	50	80	69.0
Dentist 2.	46	84	71.5
Dentist 3.	79	95	72.8
Dentist 4.	41	60	74.8
Dentist 5.	73	105	63.4
Average (N)	57.80	84.80	70.30
SD (N)	17.05	16.96	4.41

## Data Availability

Data are contained within the article or Supplementary Materials.

## Supplementary Materials

All of the necessary files to reproduce the experiments are available online under the following URL: https://data.mendeley.com/datasets/64sjwzcfz9/2. The files are in *.stl and .stp* format. The dataset contains the files to reproduce the tooth model, as well as the flexural test addons. The 3D model of the forceps is available upon request from the corresponding author.

## Appendix A

**Table A1:** Likert scale; summary of the results of the simulation of tooth extraction.

		Dentist 1	Dentist 2	Dentist 3	Dentist 4	Dentist 5	AVG
Metal Upper Right Molar Forceps	How firmly could you grab the 3D-printed tooth with the metal dental forceps?	5	4	4	4	5	4.4
	How similar was the torque you could use to what is necessary in a clinical environment?	5	4	4	4	4	4.2
	How similar was the axial extraction force you could use to what is necessary in a clinical environment?	5	5	4	4	5	4.6
Composite Upper Right Molar Forceps	How firmly could you place the forceps on the model?	3	4	4	4	3	3.6
	How could you compare the similarity of the torque you used to what is necessary in a clinical environment?	3	3	4	4	3	3.4
	How could you compare the similarity of the axial extraction force you used to what is necessary in a clinical environment?	3	4	3	4	4	3.6
	To what degree could the flexibility of the forceps influence the proper positioning of the equipment during extraction?	4	4	5	3	4	4.0
	How convenient did you find the composite forceps during the procedure?	4	5	4	5	4	4.4
	Would you use such forceps during patient care?	No	Yes	No	Yes	No	40%
	If you would not use our composite forceps in patient care, could it be made appropriate for you with some modifications?	Yes	Yes	Yes	Yes	Yes	100%
